# Comparison of clinical outcomes of toric intraocular lens, Precizon vs Tecnis: a single center randomized controlled trial

**DOI:** 10.1186/s12886-018-0955-3

**Published:** 2018-11-09

**Authors:** Na Yeon Jung, Dong Hui Lim, Sung Soon Hwang, Joo Hyun, Tae-Young Chung

**Affiliations:** 1 Department of Ophthalmology, Samsung Medical Center, Sungkyunkwan University School of Medicine, #81 Irwon-ro, Gangnam-gu, Seoul, 06351 Korea; 2 Department of Preventive Medicine, Graduate School, The Catholic University of Korea, Seoul, Korea; 3 Department of Ophthalmology, Saevit Eye Hospital, Goyang, Korea

**Keywords:** Precizon, Tecnis, Astigmatism, Toric, Intraocular Lens

## Abstract

**Background:**

To compare the clinical outcome of Precizon toric intraocular lens (IOL) (Ophtec Inc.) to that of Tecnis toric IOL (Abbott Medical Optics Inc.).

**Methods:**

This randomized comparative study included 40 eyes (Precizon, 20 eyes; Tecnis, 20 eyes) of 40 patients with visually significant cataract and corneal astigmatism who underwent cataract surgery. Changes in uncorrected distant visual acuity (UCDVA), best corrected distant visual acuity (BCDVA), uncorrected intermediate visual acuity (UCIVA), refraction, residual astigmatism, rotation of the IOL axis, and higher order aberrations at 3 months postoperatively were evaluated. Vector analysis was performed using the Alpins method.

**Results:**

Both groups showed significant reduction in refractive astigmatism after the surgery (Precizon: − 1.06 ± 0.94 Diopter (D) to − 0.31 ± 0.29 D, *p* = 0.042; Tecnis: − 1.83 ± 1.29 D to − 0.41 ± 0.33 D, *p* = 0.015). There was no significant (*p* > 0.05) difference in postoperative UCDVA, BCDVA, or residual astigmatism between the two groups, although a tendency of better UCIVA was observed in the Precizon group. Vector analysis parameters showed no statistically significant difference beween groups(*P* > 0.05). Significant difference in rotation of toric IOL axis was found between the two groups (Precizon: 1.50° ± 0.84, Tecnis: 2.56° ± 0.68, *p =* 0.010). Spherical aberration in the Precizon group was significantly (*p* = 0.005) lower than that in the Tecnis group.

**Conclusions:**

The Precizon toric IOL group had better rotational stability at 3-month postoperatively. Both Precizon toric IOL and Tecnis toric IOL could be effectively used by cataract surgeons to correct preexisting corneal astigmatism through cataract surgery.

**Trial registration:**

http://clinicaltrials.gov, NCT03085901, retrospectively registered on 21 March 2017.

**Electronic supplementary material:**

The online version of this article (10.1186/s12886-018-0955-3) contains supplementary material, which is available to authorized users.

## Background

Approximately 40 to 45% of patients who have undergone cataract surgery have more than 1 diopter (D) of corneal astigmatism [1, 2]. Toric intraocular lenses (IOLs) are becoming more commonly available, allowing more improvement in clinical outcomes than other treatment options to correct corneal astigmatism during or after cataract surgery [3]. However, if unintended rotation of one degree from the target axis of toric IOL occurs, it can result in a loss of approximately 3.3% of cylindrical power [4].

**Fig. 1:**
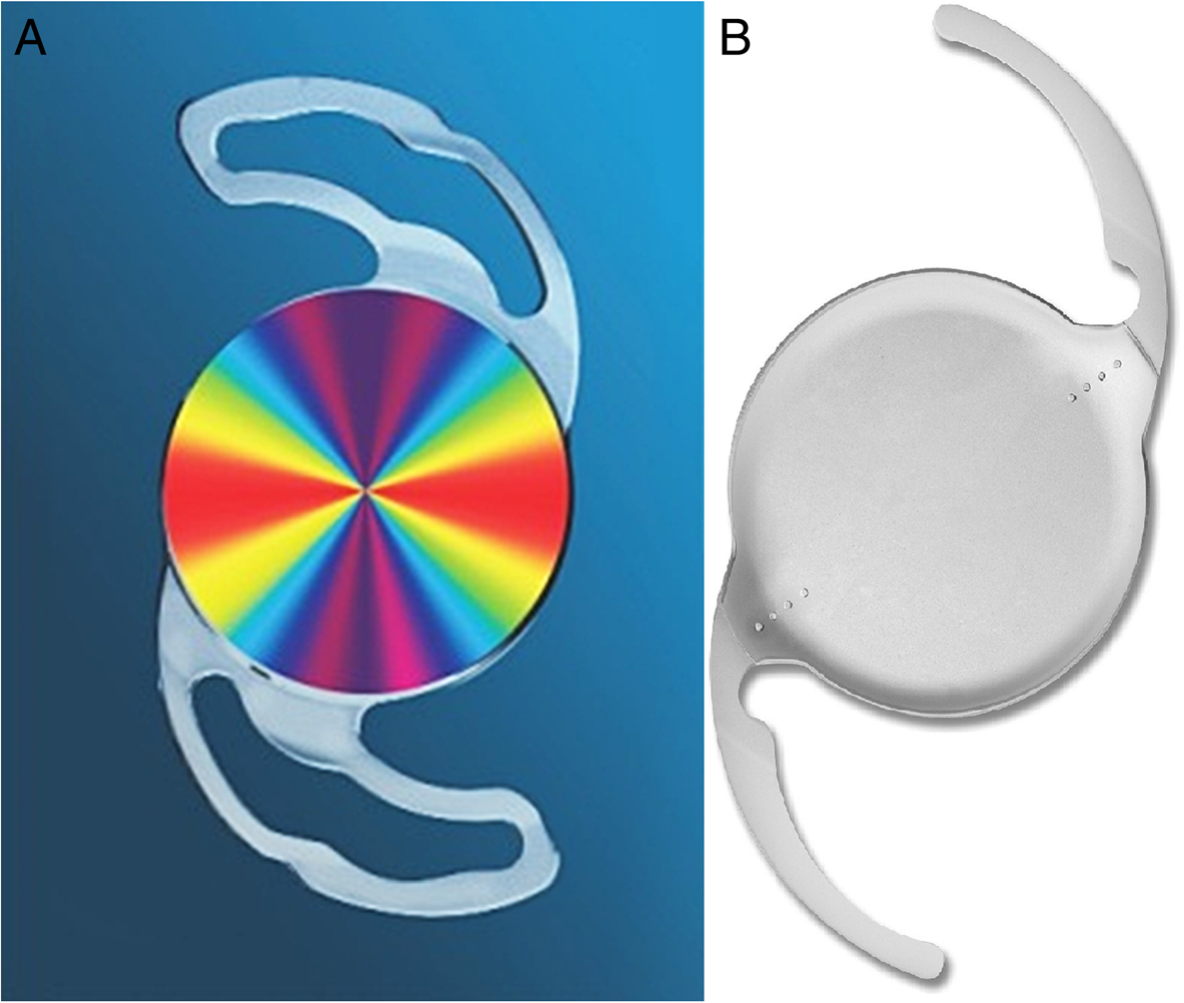
Schematic images of the toric intraocualr lens. **a**: Precizon toric intraocular lens. **b**: Tecnis toric intraocular lens (**b**)

With growing interests in reducing undesirable residual astigmatism, several ideas have been suggested for the design of toric IOLs. Precizon toric IOL (Ophtec Inc., Netherlands), one of the relatively recently introduced toric IOL, is expected to have greater resistance in postoperative rotation due to its unique optic design (Fig. 1). However, only a few clinical results have been published on this aberration free toric IOL [5–7]. Therefore the aim of this study was to evaluate the clinical outcomes of Precizon toric IOL compared to commonly used Tecnis toric IOL (Abbott Medical Optics Inc., Santa Ana, CA, USA) which has different characteristics in IOL after cataract surgery in patients with corneal astigmatism.

## Methods

### Patients selection

This prospective randomized comparative study included 40 eyes of 40 patients who were scheduled for cataract surgery with implantation of toric IOL from April 2016 at Samsung Medical Center, Seoul, Korea. Written informed consents were obtained from 40 consecutive patients before performing the study. These patients were randomly allocated into two groups to receive either Precizon toric IOL or Tecnis toric IOL during the cataract surgery. Inclusion criteria were visually significant cataract and regular corneal astigmatism measured with Scheimpflug imaging (Pentacam HR, Oculus, Wetzlar, Germany) between 0.50 diopter (D) and 2.50 D considering both anterior and posterior corneal surface. The surgically induced astigmatism (SIA) of the operating surgeon was found as 0.50 D for temporal clear corneal incision, so patients were included if their requiring corneal astigmatism correction considering surgically induced astigmatism was more than 1 D considering their steep axis. Each patients had a complete ophthalmological examination. Exclusion criteria were amblyopia, irregular astigmatism, corneal opacity, glaucoma, retinal disease, history of ocular inflammation, history of ocular trauma, and previous other intraocular surgery. Also patients were excluded if they take medications such as a-blocker. This study was approved by Institutional Review Board of Samsung Medical Center(Permission number: SMC 2016–04-147). It was carried out in accordance with the Declaration of Helsinki. The manuscript reporting adheres to the CONSORT guidelines for the reporting of randomized trials. In South Korea, clinical trial registration is not mandatorily required for these randomized comparative study. However we registered the trial in 2017 when we submit the paper to meet the international guidelines. The authors confirm that all ongoing and related trials for this drug/intervention are registered. The enrolled eyes were randomized into two groups using a computer generated random number table with a 1:1 ratio at screening visit. One investigator (N.Y.J.) generated and implemented the randomization allocation process.

### Preoperative evaluation

Preoperatively, all patients underwent complete ophthalmic evaluation including uncorrected distant visual acuity (UCDVA), best corrected distant visual acuity (BCDVA), refractive errors, and corneal topography using Scheimpflug imaging (Pentacam HR, Oculus, Wetzlar, Germany).

Biometry measurements (axial length and anterior chamber depth) used for IOL power calculation were obtained with optical coherence biometry (IOLMaster, software version 5.02, Carl Zeiss Meditec AG, Jena, Germany). The spherical power of the IOL was calculated using SRK-T formula. Emmetropia was target postoperative spherical equivalent (SE). Surgeon used his typical surgical induced astigmatism magnitude, and all main wound incision was planned to be at temporal side of the cornea. Total corneal astigmatism was calculated considering both anterior and posterior corneal surface measured with Pentacam HR using vector summation according to Alpins’ method [8]. From these data, calculations of the cylindrical power and axis placement were performed using each IOL manufacturer’s online calculator. For Precizon toric IOL, PRECIZON Online Calculator (available from: http://calculator.ophtec.com/) was used with A-constant of 118.5. For Tecnis toric IOL, Tecnis toric express calculator (available from: http://www.amoeasy.com/calc/) was used with A-constant of 119.3. Surgically induced astigmatism of 0.50 D was assumed for all cases.

### Intraocular lenses

Characteristics of both IOLs are shown in Additional file 1: Table S1. The Precizon toric IOL Model 565 (Ophtec BV) is a piece of hydrophilic acrylic, monofocal, and aspheric IOL with a transitional conic toric surface (patent pending). It has consistent power from the center to the periphery, yielding a broader toric meridian. It is more resistant of IOL misalignment [9]. It has a closed-loop haptic design. This lens is also aberration free. Tecnis toric IOL has anterior toric surface with a proprietary wavefront-designed toric aspheric optic, resulting in negative spherical aberration [10]. It has open-loop C-haptics. Both toric IOL have two reference marks on the axis of the cylinder of their surface.

### Surgical technique

Before surgery, 0° - 180° axis was marked with all patient seating upright at slit-lamp using a horizontal slit beam. Intraopertively, intended implantation axis was marked on the limbus after correctly aligning a Mendez ring to the primary marks to ascertain the intended angle of placement according to preoperative plan. One experienced surgeon (T.Y.C.) performed all surgeries under topical anesthesia (proparacaine hydrochloride 0.5%, Alcaine; Alcon Laboratories, Fort Worth, TX, USA). Phacoemulsification was performed through a 2.75 mm temporal clear corneal incision. After performing continuous curvilinear capsulorrhexis with an intended diameter 5.0 mm and hydrodissection, phacoemulsification of the nucleus and bimanual aspiration of the residual cortex were conducted using Centurion Vision System (Alcon, Laboratories, Fort Worth, TX, USA). Toric IOL was implanted in the capsular bag using injector and disposable cartridge system before removing ophthalmic viscosurgical device (OVD). After removing the OVD, the IOL was rotated to its final targeted position by exactly aligning the toric reference marks on the IOL surface with limbal axis marks. Finally, a balanced salt solution was injected into the incision site to close the corneal incision, causing edema. Before finishing the surgery, intraoperative photographs were taken for all cases. After the surgery, postoperative eye drops of antibiotics (gatifloxacin 0.3%, Gatiflo; Handok, Seoul, Korea) and corticosteroid (lotepredrol etabonate, lotemax; Bausch + Lomb, Tampa, FL, USA) were used 4 times a day. They were tapered over a month. For all patients, non-steroidal anti-inflammatory drugs (NSAIDs) (ketorolac tromethamine 0.45%, Ocuveil; Allergan, Inc., Irvine, CA, USA) were used for 2 weeks.

### Postoperative evaluation

Postoperative examinations were performed at 1 day, 1 week, 1 month, and 3 months after the surgery. All patients underwent measurement of UCDVA, BCDVA, uncorrected intermediate (80 cm) visual acuity (UCIVA), manifest refraction, and slit-lamp examination with IOP measurement. At 1-month and 3-month postoperatively, ocular wavefront aberrometry was performed using WASCA (Carl Zeiss Meditec AG, Jena, Germany). Parameters analyzed for a 5.0 mm pupil included vertical and horizontal coma, vertical and horizontal trefoil, spherical aberration, and root mean square (RMS) values of total aberrations and high order aberrations. The WASCA abberometer provided Zernike coefficients in Malacara notation. However, results are presented in standard notation of Optical Society of America(OSA).

### Vector analysis

Vector analysis was performed using the Alpins method, facilitated by the ASSORT program version 5.04 (Assort Pty., Ltd., Victoria, Australia). Target induced astigmatism (TIA) was defined as the astigmatic change in the magnitude and axis the surgery was intended to correct. Therefore, actual measured preoperative corneal topographic astigmatism was used. Surgically induced astigmatism (SIA) was defined as the amount and axis of the astigmatism the surgery actually induced. Difference vector was defined as the induced astigmatic change by the magnitude and axis that would enable the initial surgery to achieve its intended astigmatic target. That means the difference vector is the actual measured postoperative refraction remaining after the surgery. Correction index calculated by determining ratio of SIA to TIA (correction index is preferably 1.0; if correction index > 1.0 overcorrection occurred and if correction index < 1.0 undercorrection occurred). Magnitude of error is the arithmetic difference between magnitudes of SIA and TIA (magnitude of error > 0 indicates overcorrection and magnitude of error < 0 undercorrection). Angle of error is the angle described by the vectors of SIA versus TIA (angle of error > 0: achieved correction index is counterclockwise to where it was intended; angle of error < 0: achieved correction is clockwise to its intended axis). Index of success is calculated by dividing the difference vector by TIA, representing a relative measure of success (index of success is preferably 0).

### Rotational stability analysis

Rotations of the IOL were assessed by analyzing digital photographs in retro-illumination of the IOL with full mydriasis. Conjunctival vessels, iris patterns, or conjunctival pigmented lesions were selected as a reference point to compare the axis between photographs. Postoperative rotation was defined as the difference between intraoperative axis and the achieved axis at 3 months postoperatively. The absolute rotation amount was analyzed by calculating differences between the angle of the IOL reference marks of intraoperative photographs and 3 months postoperative photographs using ImageJ program. One independent investigator performed the measurement.

### Sample size

The study population was calculated according to previous conducted studies, Vale et al. [5] and Sheppard AL et al. [10] assuming 1:1 randomization with a significance level of 5% and a power of 80%. Based on previous data, the sample size was calculated to be 40 eyes were required, corresponding to 20 eyes in each group.

### Statistical analysis

All data were inputted into Excel database (Microsoft Corporation, Redmond, WA, USA). Statistical analyses were performed using SPSS software system for Windows, Version 20 (SPSS Inc., Chicago, IL). Visual acuities were converted into logMAR for mathematical and statistical calculations. Paired t test was used to compare visual acuity and refractive parameters between preoperative and postoperative examinations. Independent t test was used for between-group comparisons. Results are expressed as means ± standard deviation of the means. Statistically significance was considered when *P* value was less than 0.05.

## Results

Based on our study protocol, 40 eyes from 40 patients aged between 22 and 87 years were included in this study. Patient recruitment was from April 2016 to July 2016. The study was finished after 3 months postoperative follow up visit was completed for all patients in October 2016. The Precizon group included 20 eyes from 20 patients. The Tecnis group included 20 eyes from 20 patients. All patients received regular follow-up examinations for at least 3 months. Patients’ demographics and IOL models used in the two groups are summarized in Table 1. Preoperatively, there was no significant (*P* > 0.05) difference between the two groups.

**Table 1:** Demographics and clinical information of patients included in this study

	Precizon	Tecnis	*p* value
Eyes (n)	20	20	
Patients (n)	20	20	
Age (y)	64.64 ± 19.55 (22.5 to 87.1)	64.51 ± 8.40 (46.6 to 87.8)	0.980
Male sex, n (%)	8 (40)	11 (55)	0.527
Right eyes, n (%)	8 (40)	10 (50)	0.751
UCDVA (logMAR)	0.50 ± 0.17 (0.30 to 0.82)	0.37 ± 0.13 (0.22 to 0.52)	0.123
BCDVA (logMAR)	0.30 ± 0.18 (0 to 0.7)	0.21 ± 0.13 (0 to 0.4)	0.266
Manifest Refraction			
Sphere (D)	−0.12 ± 1.32 (−1.75 to 2.75)	0.64 ± 2.82 (− 6.00 to 3.75)	0.418
Cylinder (D)	− 1.06 ± 0.94 (− 2.50 to − 0.50)	− 1.83 ± 1.29 (− 4.00 to − 0.50)	0.129
SE (D)	−0.66 ± 1.47 (− 2.50 to 2.75)	−0.28 ± 2.64 (− 6.50 to 1.88)	0.680
Corneal astigmatism (D)	1.32 ± 0.45 (0.53 to 2.15)	1.47 ± 0.47 (0.72 to 2.09)	0.465
IOL power (D)	19.56 ± 2.35 (15.75 to 23.75)	20.11 ± 3.52 (16.00 to 25.50)	0.629
IOL Cylinder power (D)	1.96 ± 0.84 (1.00 to 3.50)	2.36 ± 0.76 (1.50 to 4.00)	0.266
Axial length (mm)	23.86 ± 1.04 (22.01 to 25.57)	24.22 ± 0.83 (23.15 to 25.22)	0.387

Mean ± SD (range)

*Y* years, *LogMAR* Logarithm of the minimum angle of resolution, *D* diopter, *UCDVA* uncorrected distance visual acuity, *BCDVA* best corrected distance visual acuity, *IOL* intraocular lens, *SE* spherical equivalent refraction

### Visual acuity and refraction

After cataract surgery, UCDVA, BCDVA, and cylindrical errors were significantly (*P* < 0.05) improved in both groups (Table 2). In the Precizon group, UCDVA was significantly (*P* < 0.05) increased from 0.50 ± 0.17 (range, 0.30 to 0.82) logMAR preoperatively to 0.09 ± 0.09 (range, 0 to 0.30) logMAR after 3 months postoperatively. In the Tecnis group, UCDVA was also significantly (*P* < 0.05) improved from 0.38 ± 0.13 (range, 0.22 to 0.52) logMAR preoperatively to 0.08 ± 0.12 (range, 0 to 0.30) logMAR at 3 months postoperatively. The percentage of UCDVA that was 0.1 logMAR or better (Snellen chart 20/25 or better) was 91% in the Precizon group and 83% in the Tecnis group.

**Table 2:** Preoperative and postoperative clinical data in the Precizon toric intraocular lens group and Tecnis toric intraocular lens group at 3-month postoperatively

Parameters	Precizon			Tecnis			*P* ^*^ value
	Preop	Postop	*p* value	Preop	Postop	*p* value	
UCDVA (logMAR)	0.50 ± 0.17 (0.30 to 0.82)	0.09 ± 0.09 (0 to 0.30)	*0.005*	0.38 ± 0.13 (0.22 to 0.52)	0.08 ± 0.12 (0 to 0.30)	*0.020*	0.904
BCDVA (logMAR)	0.30 ± 0.18 (0 to 0.7)	0.02 ± 0.02 (0 to 0.05)	*0.008*	0.21 ± 0.13 (0 to 0.50)	0.01 ± 0.02 (0 to 0.05)	*0.042*	0.582
UCIVA (logMAR)	No data	0.26 ± 0.13 (0.09 to 0.49)		No data	0.40 ± 0.16 (0.20 to 0.60)		0.114
Manifest Refraction							
Sphere (D)	−0.12 ± 1.32 (− 1.75 to 2.75)	0.25 ± 0.35 (− 0.25 to 1.00)	0.324	0.64 ± 2.82 (− 6.00 to 3.75)	0.19 ± 0.48 (− 0.25 to 1.25)	0.260	0.753
Cylinder (D)	−1.06 ± 0.94 (− 2.50 to − 0.50)	−0.31 ± 0.29 (− 0.75 to 0)	*0.042*	−1.83 ± 1.29 (− 4.00 to − 0.50)	−0.41 ± 0.33 (− 0.75 to 0)	*0.015*	0.491
SE (D)	− 0.65 ± 1.47 (− 2.50 to 2.75)	0.06 ± 0.38 (− 0.50 to 0.75)	0.184	−0.28 ± 2.64 (− 6.50 to 1.88)	−0.04 ± 0.50 (− 0.63 to 1.00)	0.726	0.600
Rotation (°)	No data	1.50 ± 0.84 (0.18 to 3.02)		No data	2.56 ± 0.68(1.50 to 3.50)		*0.012*

Mean ± SD (range)

*SD* standard deviation, *UCDVA* uncorrected distance visual acuity, *LogMAR* Logarithm of the minimum angle of resolution, *BCDVA* best corrected distance visual acuity, *UCIVA* uncorrected intermediate visual acuity, *D* diopter, *SE* spherical equivalent refraction

*P values between the two groups, *P* < 0.05

In the Precizon group, BCDVA was significantly (*P* < 0.05) increased from 0.30 ± 0.18 (range, 0 to 0.70) logMAR preoperatively to 0.02 ± 0.02 (range, 0 to 0.05) logMAR at 3 months postoperatively. In the Tecnis group, BCDVA was also significantly (*P* < 0.05) improved from 0.21 ± 0.13 (range: 0 to 0.50) logMAR preoperatively to 0.01 ± 0.02 (range, 0 to 0.05) logMAR at 3 months postoperatively (Table 2). The final BCDVA of all eyes in both groups achieved 0.05 logMAR (Snellen chart 20/25 or better).

The refractive cylinder was decreased from − 1.06 ± 0.94 D preoperatively to − 0.31 ± 0.29 D (70% decrease) at 3 months postoperatively in the Precizon group. It was decreased from − 1.83 ± 1.29 D preoperatively to − 0.41 ± 0.33 D (77% decrease) at 3 months postoperatively in the Tecnis group (Table 2). At the last follow-up, residual refractive cylinder which was less than 0.50 D occurred in 16 (80%) eyes in the Precizon group and in 14 (70%) eyes in the Tecnis group.

Results of postoperative visual acuity and refraction in both groups are shown in Table 2. No statistically significant difference in UCDVA or BCDVA (*P* = 0.562, *P* = 0.368, respectively) was found between the two groups. UCIVA in the Precizon group tended to be better compared to that of the Tecnis group. However, the difference was not statistically significant (*P* = 0.147). No significant difference in refractive outcomes (sphere, cylinder, and spherical equivalent, *P* = 0.423, *P* = 0.604, and *P* = 0.400, respectively) was found between the two groups.

### Vector analysis

Vector anlysis was pereformed at 3 months postoperatively (Table 3). The TIA vector means were 1.41 ± 0.49 D in the Precizon group and 1.41 ± 0.43 D in the Tecnis group.

**Table 3:** Vector Analysis of astigmatism at 3-Month Postoperatively

Parameters	Mean ± SD (range)		*p* value
	Precizon	Tecnis	
TIA (D)	1.41 ± 0.49 (0.54 to 2.30)	1.41 ± 0.43 (0.75 to 2.18)	0.982
SIA (D)	1.35 ± 0.52 (0.53 to 2.15)	1.57 ± 0.79 (0.70 to 2.84)	0.468
DV (D)	0.31 ± 0.23 (0.01 to 0.76)	0.42 ± 0.26 (0.01 to 0.82)	0.343
Correction index (SIA/TIA)	0.97 ± 0.25 (0.62 to 1.45)	1.08 ± 0.27 (0.67 to 1.49)	0.377
Magnitude of error (arithematic SIA/TIA)	−0.06 ± 0.34 (− 0.58 to 0.46)	0.16 ± 0.45 (− 0.45 to 0.81)	0.233
Angle of error (degree)	0.19 ± 4.80 (− 6.7 to 14.4)	−2.78 ± 6.71 (− 20.4 to 1.6)	0.274
Absolute angle of error (degree)	2.42 ± 4.10 (0.00 to 14.4)	3.18 ± 6.52 (0.10 to 20.40)	0.761
Index of success (DV/TIA)	0.23 ± 0.20 (0.02 to 0.75)	0.30 ± 0.20 (0.01 to 0.68)	0.470

*SD* standard deviation, *TIA* target induced astigmatism, *SIA* Surgically induced astigmatism, *DV* difference vector

No statistically significant difference in average TIA vector nor average SIA vector (*P* = 0.982, *P* = 0.468, respectively) was found between the two groups. The average DV for the Precizon and Tecnis groups were 0.31 ± 0.23 versus 0.42 ± 0.24, respectively, and these were not significantly different (*P* = 0.343). The mean correction index (ratio SIA to TIA; preferably 1), were 0.97 ± 0.25 versus 1.08 ± 0.27, respectively, reflecting slight undercorrection in the Precizon group and slight overcorrection in the Tecnis group(*P* = 0.377). Other vector analysis parameters show no statistically significant difference beween groups(*P* > 0.05).

### Rotational stability

The mean amount of toric IOL axis rotation was 1.50° ± 0.84° (range, 0.18° to 3.02°) in the Precizon group, which was significantly (*P* = 0.01) lower than that (2.56° ± 0.68°; range, 1.50° to 3.50°) in the Tecnis group (Table 2). No eye had IOL rotation for more than 4°. No eye required a second surgery to correct the IOL axis during the 3 months of follow-up period.

### Ocular wavefront aberration

Ocular wavefront aberrometry values at 3 months postoperatively are shown in Table 4. Spherical aberration was significantly (*P* = 0.004) lower in the Tecnis group compared to that in the Precizon group. Other ocular wavefront aberrametry parameters showed no statistically significant (*P* > 0.05) difference between the two groups.

**Table 4:** Ocular Aberrometry Analysis at 3-Month Postoperatively

Parameters	Mean ± SD (range)		*p* value
	Precizon	Tecnis	
Total Aberrations RMS (μm)	1.39 ± 0.86 (0.50 to 3.08)	0.78 ± 0.53 (0.24 to 1.67)	0.138
HOA RMS(μm)	0.37 ± 0.15(0.20 to 0.76)	0.22 ± 0.11 (0.10 to 0.38)	0.200
Vertical coma (μm)	0.07 ± 0.23 (− 0.22 to 0.47)	−0.05 ± 0.18 (− 0.30 to 0.23)	0.290
Horizontal coma (μm)	0.01 ± 0.73 (− 1.94 to 0.60)	−0.10 ± 0.73 (− 1.40 to 0.59)	0.757
Vertical trefoil (μm)	−0.01 ± 0.33 (− 0.47 to 0.50)	0.10 ± 0.24 (− 0.16 to 0.49)	0.495
Horizontal trefoil (μm)	0.37 ± 0.54 (− 0.63 to 1.02)	0.10 ± 0.67 (− 0.99 to 0.79)	0.412
Spherical aberration (μm)	0.33 ± 0.16 (0.18 to 0.68)	0.06 ± 0.16 (− 0.19 to 0.30)	*0.004*

*SD* standard deviation, *RMS* root mean square, *HOA* higher order aberrations Malacara notation was converted to Optical Society of America standard notation, *P* < 0.05

## Discussion

Using toric IOLs to correct corneal astigmatism at the time of cataract surgery has greatly improved both postoperative visual performance and satisfaction of the patient [3, 11]. Many IOLs are available with different characteristics. They are designed to improve the clinical outcomes including visual acuity, correction of astigmatism, and rotational stability.

**Fig. 2:**
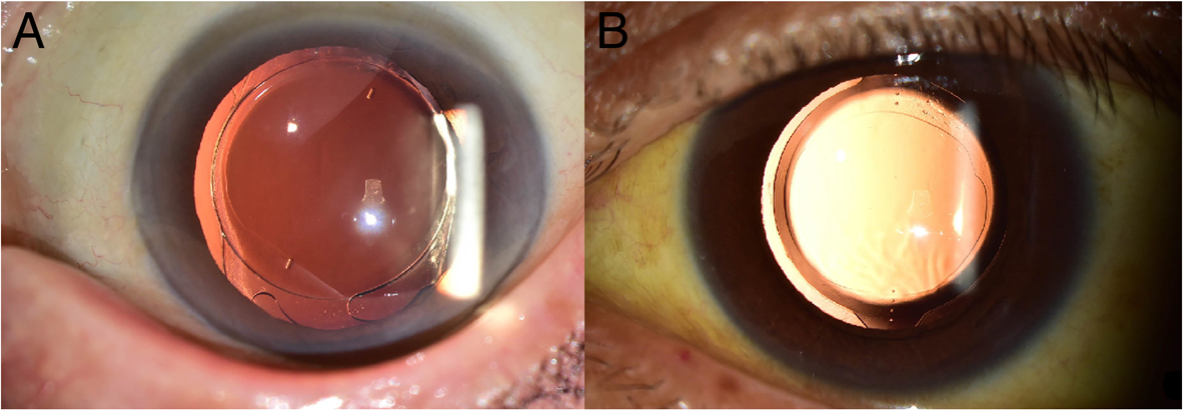
Slit lamp images of the toric intraocular lens at 3 months postoperatively. **a**: Precizon toric intraocular lens. **b**: Tecnis toric intraocular lens. The axis markings can be clearly seen

Although several studies have reported the clinical outcomes of different toric IOLs, to the best of our knowledge, Precizon toric IOL compared to other toric IOL has not been reported yet. Precizon toric IOL is a relatively recently introduced toric IOL. It has been reported that Precizon toric IOL is more resistant to reduction of astigmatic correcting effects because of its unique toric surface of conic design when unexpected IOL rotation occurs [9]. Due to its transitional conic toric surface, Precizon toric IOL has consistent toric power from the center to the periphery, yielding a broader toric meridian (Fig. 1a). Therefore, Precizon toric IOL is expected to more tolerable to postoperative rotation [9]. In an optical bench analsysis, precizon’s transitional conic toric surface demonstrated maximal rotational resistance compared with other toric IOL models (AT Torbi 709, SN6AT4, ZCT 225) [12]. Together with AT Torbi Precizon also showed superior image quality despite the pupil size changes in the presence of decenteration [12]. Our aim was to determine the clinical outcomes of Precizon toric IOL in comparison with Tecnis toric IOL, a commonly used IOL. Precizon toric IOL has a closed-loop haptic design. This lens is also aberration free. Tecnis toric IOL has open-loop C haptics (Fig. 1b). It has anterior toric surface with a proprietary wavefront-designed toric aspheric optic, resulting in negative spherical aberration [10]. Both toric IOL have two reference marks on the axis of the cylinder of their surface (Fig 2). Because of their definite differences in IOL characteristics, we hypothesized that different clinical results would be obtained for the two groups. However, their postoperative clinical results for many parameters were similar to each other, except postoperative IOL rotation.

UCDVA is one of the most important parameters used to determine the success in patients who undergo the surgery. In our study, the average UCDVA was 0.09 ± 0.09 logMAR in the Precizon group and 0.08 ± 0.12 logMAR in the Tecnis group. The percentage of patients who achieved 0.1 logMAR (Snellen chart 20/25 or better) was 91% in the Precizon group and 83% in the Tecnis group. BCDVA of all eyes achieved 0.05 logMAR (Snellen acuity 20/22 or better) in both groups. Vale et al. [5] have reported that 100% of eyes have achieved a UCDVA of 0.20 logMAR (Snellen chart 20/30 or better) and Ferreira et al. [6] have reported that 82% of eyes have achieved a UCDVA of 0.10 logMAR (Snellen chart 20/25 or better) when Precizon toric IOL is used. Lubinski et al. [13] have also reported that all eyes have achieved 0.30 logMAR (Snellen chart 20/40 or better) when Tecnis toric IOL is used, similar to the outcome of the Tecnis toric IOL group in this study.

Rocha et al. [14] have shown that near and intermediate visual acuities are better in eyes with spherical IOLs compared to those with aspheric IOLs. They have concluded that residual spherical aberration can improve the depth of focus. Johansson et al. [15] have also found the depth of focus in aberration free IOLs is increased compared to negative spherical aberration IOLs. In our study, different toric IOLs (spherical aberration free and negative spherical aberration) were implanted to determine the difference in visual acuity between the groups. We planned to measure the visual acuity at 80 cm intermediate distance in postoperative evaluation because spherical aberration could increase the depth of focus [15, 16]. Although all parameters of visual acuity showed no significant difference between the two groups in this present study, UCIVA in the Precizon group did show a tendency to be better than that in the Tecnis group. The result of no significant difference in intermediate visual acuity between the two groups after the surgery might be due to the fact that the proportion of patients with large preoperative corneal astigmatism was relatively small in our sample size compared to previous studies.

The residual refractive cylinder at 3 months postoperatively was − 0.31 ± 0.29 D in the Precizon group, similar to the result of previous studies, Vale et al. [5] (0.27 ± 0.28 D at 6 months postoperatively), Ferreira et al. [6] (− 0.51 ± 0.29 D at 4 months postoperatively) and Thomas et al. [7] (− 0.25 D at 3 months postoperatively). The residual refractive cylinder in the Tecnis group was − 0.41 ± 0.33 D at 3 months postoperatively, which was smaller than − 0.56 ± 0.35 D at 8 weeks postoperatively or − 1.42 ± 0.88 D at 6 months postoperatively reported in previous studies [10, 13]. Because we considered both anterior and posterior corneal astigmatism, whereas other previous studies only considered anterior corneal astigmatism, we expected to have much smaller residual cylinder in both IOLs. However, the residual refractive cylinder in the Precizon group in our study was similar to that of a previous studies [5–7]. This might be due to the fact that preoperative corneal astigmatism in our study (1.32 ± 0.48 D) was much smaller than that in the previous studies, Vale et al. [5] (2.34 ± 0.95 D), Ferreira et al. [6] (2.38 ± 1.17 D) and Thomas et al. [7] (1.50 D). Subject component of the refraction and the effect of corneal incision might have also contributed to such result. Also the differences in postoperatve evaluation time between the studies might be the factor.

In the current study we analyzed the astigmatic change using Alpins method. No statistically significant difference in average TIA vector nor average SIA vector (*P* = 0.982, *P* = 0.468, respectively) was found between the two groups. The average DV for the Precizon and Tecnis groups were 0.31 ± 0.23 versus 0.42 ± 0.24, respectively, and these were not significantly different (*P* = 0.343). The mean correction index (ratio SIA to TIA; preferably 1), were 0.97 ± 0.25 versus 1.08 ± 0.27, respectively, reflecting slight undercorrection in the Precizon group and slight overcorrection in the Tecnis group, but there was no statistcally significant difference between two groups (*P* = 0.377). Also the Magnitude of error (arithmetic ration SIA to TIA: magnitude of error > 0 indicates overcorrection and magnitude of error < 0 undercorrection), were − 0.06 ± 0.34 versus 0.16 ± 0.45, respectively, which showed tendency of slight undercorrection in the Precizon group and slight overcorrection in the Tecnis group with no significant differences. In the previous study, Vale et al. [5] have also reported the mean difference vector as 0.24 ± 0.27, mean correction index as 0.95 ± 0.29, Absolute angle of error as 1.90 ± 0.60 (degree), and mean index of success as 0.12 ± 0.14, respectively. Kirwan C et al. [17] reported difference vector as 0.93 and correction index ratio as 1.17. There were few datas which conducted vector analysis using alpins method of the Precizon toric IOL and Tecnis toric IOL. Although the comparing with few previous data has some limitation, vector analysis of astigmatic change showed both two toric IOLs showed effective astigmatic correction.

Total aberration RMS, HOA RMS, coma, trefoil showed no significant difference between Precizon and Tecnis IOL in this current study. Spherical aberration was significantly higher in the Precizon IOL group(*P* = 0.004). The Tecnis IOL has − 0.27 um spherical aberration and the Precizon IOL is aberration free IOL. Our results means both IOLs realized its aspheric feature. Residual spherical aberration could improve the depth of focus, and it can help the near and intermediated vision in certain part.

The main postoperative complication after implantation of toric IOLs might be rotation. It has been estimated that a rotation of 1 degree off axis can result in a loss of up to 3.3% of IOL cylinder power [4]. When misalignment is greater than 30 degrees, there might be no correction effect on the astigmatism and a shift in resultant astigmatic axis might occur. The rotational stability of the Precizon toric IOL in our study was significantly better than that in the Tecnis toric IOL group. Vale et al. [5], Ferreira et al. [6] and Thomas et al. [7] have reported that the rotation of the Precizon toric IOL was about 2.43° ± 1.55°, 1.98° ± 1.78° and 3°, respectively. Wasltz KL et al. [18] have found that the postoperative IOL rotation is 2.70° ± 5.51° when Tecnis toric IOL is used, while Ferreira TB et al. [19] and Yang et al. [20] have reported postoperative IOL rotation of 3.15° ± 2.62° and 3.2° ± 2.2°, respectively. In the present study, the absolute amount of postoperative rotation was 1.50° ± 0.84° (range, 0.18° to 3.02°) in the Precizon group and 2.56° ± 0.68° (range, 1.50° to 3.50°) in the Tecnis group, which were smaller than those reported previously for the two toric IOLs. Although there was a definite difference in the IOL design of the two toric IOLs (we also found significant less IOL rotation in the Precizon group compared to that in the Tecnis group), no significantly difference in clinical outcomes in terms of astigmatism correction of visual acuities at 3 months postoperatively was found between the two groups. Therefore, we conclude that such difference in rotational stability between the two groups might have minor clinical importance due to their small numerical amounts in both groups. Future studies with more participants of greater corneal astigmatism and longer postoperative follow up period are needed to determine whether the two will show different outcomes. In addition, if greater amount of rotation of IOL occurs, clinical result might show significant difference in further studies.

There are some limitations of this study. Every study patient had undergone compelete ophthalmic examination before the surgery. If the patient showed asymmetry of capsular bag or absence of the zonules, they were excluded for study. No ocular adverse event occurred during the study. However, differences of capsular bag diameter between two groups might affect the differences in the rotational stability of the toric IOL in some part. Future study regarding size of the capsular bag can strengthen the clinical significancy.

## Conclusions

In summary, our results showed that Precizon toric IOL was better than Tecnis toric IOL in rotational stability with follow up period of 3 months. Both Precizon toric IOL and Tecnis toric IOL appear to be effective alternatives for cataract surgeons to correct preexisting corneal astigmatism through cataract surgery.

## Additional file

Additional file 1: **Table S1.** Characteristics of the Precizon toric IOL and Tecnis toric IOL. (DOCX 26 kb)

## Abbreviations

BCDVA: Best corrected distant visual acuity
D: Diopter
IOL: Intraocular lens
LogMAR: Logarithm of the minimum angle of resolution
RMS: Root mean square
SE: Spherical equivalent
SIA: Surgically induced astigmatism
UCDVA: Uncorrected distant visual acuity
UCIVA: Uncorrected intermediate visual acuity
