# Supplementary material for: Comparison of clinical outcomes of toric intraocular lens, Precizon vs Tecnis: a single center randomized controlled trial
Source: BMC Ophthalmol. 2018 Nov 9;18:292. doi: 10.1186/s12886-018-0955-3 (PMC6230282; doi:10.1186/s12886-018-0955-3)
Supplement: Supplementary file 1 — Table S1. Characteristics of the Precizon toric IOL and Tecnis toric IOL. (DOCX 26 kb) [file 12886_2018_955_MOESM1_ESM.docx]

**S1 Table. Characteristics of the Precizon toric IOL and Tecnis toric IOL.**

| Characteristics | Precizon toric IOL | Tecnis toric IOL |
| --- | --- | --- |
| Optic characteristics |  |  |
| Powers (D) | +10 to +30 D | +5.00 to +34.00 |
| Cylinder powers (IOL plane) (D) | 1 to 10 D  (0.5 D increments) | 1.50/2.25/3.00/4.00 |
| Cylinder powers (corneal plane) (D) | 0.69 to 6.85 | 1.03/1.54/3.06/2.74 |
| Diameter (mm) | 6.0 | 6.0 |
| Shape | Biconvex, transitional conic toric aspheric surface | Biconvex, anterior toric aspheric surface |
| Material | Acrylic - hydrophilic | Acrylic - UV filtering hydrophobic |
| Refractive Index | 1.46 | 1.47 |
| Edge design | Square edge | Square edge |
| Spherical aberration ([㎛](https://search.naver.com/search.naver?where=nexearch&query=%E3%8E%9B&ie=utf8&sm=tab_she&qdt=0)) | 0 | -0.27 |
| Biometry |  |  |
| A-constant (mm) | 118.0 (US); 118.5 (optical) | 118.8 (US); 119.3 (optical) |
| Theoretical ACD (mm) | 5.26 | 5.4 |
| Haptic characteristics |  |  |
| Overall length (mm) | 12.5 | 13.0 |
| Configuration | close-loop design | modified C-loop design, integral with optic |
| Material | Same as optic | Same as optic |

IOL: intralocular lens; ACD: anterior chamber depth; US: ultrasound.
